# Functional Recovery Patterns and Exercise Tolerance in Children With Persistent Asthma: An Observational Study Using Spirometry and the Six-Minute Walk Test (6MWT)

**DOI:** 10.7759/cureus.103012

**Published:** 2026-02-05

**Authors:** Suwetha S, Aravind Devarajan, Vidhya Shiva Lakshmi

**Affiliations:** 1 Pediatrics and Child Health, Stanley Medical College, Chennai, IND; 2 General Surgery, Rajendra Institute of Medical Sciences, Ranchi, IND; 3 Surgery, District Hospital, Giridih, IND

**Keywords:** exercise tolerance, pediatric asthma, persistent asthma, six-minute walk test, spirometry

## Abstract

Background

Bronchial asthma in children is associated with progressive airway inflammation and variable functional limitation. While spirometry remains central to disease assessment, functional exercise capacity may not parallel physiological impairment. Data describing short-term functional recovery using combined spirometric and exercise-based assessment in children with asthma are limited.

Methods

This observational study with a short-term follow-up component was conducted at a tertiary care pediatric center in Chennai between April 2021 and August 2022. Children aged 6-12 years with bronchial asthma diagnosed according to Global Initiative for Asthma (GINA) criteria were enrolled. Children with asthma controlled with low-dose inhaled corticosteroids or short-acting beta-agonists as needed were categorized as having mild asthma. Those requiring medium-dose inhaled corticosteroids or long-acting beta-agonists were categorized as moderate asthma, and children with high-dose inhaled corticosteroids or long-acting beta-agonists were categorized as severe asthma. Pulmonary function was assessed using standardized spirometry in accordance with American Thoracic Society/European Respiratory Society 2019 guidelines, and functional capacity was evaluated using the six-minute walk test (6MWT). Spirometric parameters and 6MWT performance were compared across asthma severity categories using appropriate statistical tests. Children with abnormal baseline forced vital capacity (FVC) were offered repeat spirometry at three months.

Results

A total of 250 children with asthma were included, with a male-to-female ratio of 1.5:1. Spirometric impairment demonstrated a stepwise worsening with increasing asthma severity, with the most pronounced abnormalities observed in severe asthma. In contrast, six-minute walk performance remained largely preserved across severity groups. Comparative analysis showed significant differences in the distribution of abnormal FVC values between mild and severe and between moderate and severe asthma categories. Among the 57 children with abnormal baseline FVC who completed follow-up, spirometric improvement was observed in most children with mild and moderate asthma, whereas recovery was limited in those with severe asthma.

Conclusions

Spirometric impairment in children with asthma worsens progressively with disease severity, whereas functional walking capacity remains relatively preserved until later stages. These findings highlight a dissociation between physiological airflow limitation and functional exercise tolerance. Integrated monitoring strategies combining spirometry with functional assessment may provide a more comprehensive evaluation of pediatric asthma and support optimized follow-up and management.

## Introduction

Asthma is one of the most prevalent chronic respiratory illnesses in childhood and continues to exert a substantial impact on physical functioning, peer interactions, and overall quality of life [1,2]. Persistent airway inflammation, unpredictable airflow obstruction, and episodic bronchoconstriction contribute to ongoing symptoms and exercise limitation, particularly among children with moderate to severe asthma. Even when clinical symptoms appear mild, objective assessment of lung function remains essential, as spirometry can detect subclinical abnormalities that may not be evident on clinical examination. Early reductions in forced expiratory volume in one second (FEV₁) and related indices may indicate the onset of irreversible airway changes, and delays in diagnosis or inadequate disease control can further accelerate functional decline [1,2].

Spirometry remains the cornerstone for confirming the diagnosis of asthma and monitoring disease progression in children, providing objective measures of airflow obstruction, bronchodilator responsiveness, and small-airway involvement [3]. However, spirometry is underutilized in clinical pediatric practice, resulting in missed opportunities for early detection of physiological deterioration. Moreover, conventional parameters such as FEV₁ may not fully capture subtle functional impairment, prompting increasing interest in complementary measures such as mid-expiratory flow rates and serial follow-up assessments [4].

Exercise intolerance is a common manifestation of poorly controlled pediatric asthma. Increased ventilatory demand during physical activity can precipitate bronchoconstriction, shorten exercise duration, and promote activity avoidance [5]. Evidence suggests that aerobic exercise and pulmonary rehabilitation programs improve walking distance, symptom burden, and quality of life in children with asthma, even when improvements in spirometric indices are modest. Functional assessments such as the six-minute walk test (6MWT) have been shown to correlate with asthma control, small-airway function, and treatment responsiveness, particularly in moderate to severe disease [6,7]. In addition, population-specific normative data, including those derived from Indian children, demonstrate that age and sex influence functional performance, underscoring the importance of contextual interpretation [8,9].

Despite these advances, prospective studies evaluating short-term functional recovery in children with asthma through the combined use of spirometry and the 6MWT remain limited. Most existing research has focused on structured rehabilitation interventions or relied on cross-sectional comparisons. Consequently, the natural trajectory of spirometric improvement and exercise tolerance during routine guideline-based therapy, particularly within the first three months, when treatment response typically stabilizes, has not been adequately characterized. Understanding this early recovery pattern is essential for optimizing follow-up strategies and facilitating timely identification of inadequate disease control [9].

Accordingly, this study aimed to characterize three-month functional recovery patterns in children with asthma by assessing changes in spirometric indices and six-minute walk distance and by examining the relationship between pulmonary function and exercise tolerance during standard guideline-directed treatment.

## Materials and methods

This prospective observational study with a three-month follow-up was conducted at the Institute of Social Paediatrics, Government Stanley Medical College and Hospital, Chennai, between April 2021 and August 2022. Ethical approval was obtained from the Institutional Ethics Committee prior to enrollment. Written informed consent was obtained from parents or guardians, and assent was obtained from children aged more than seven years. Children aged 6-12 years were grouped for asthma severity (mild, moderate, and severe persistent) based on Global Initiative for Asthma (GINA) 2021 guidelines applicable at the time of study conduct. Children younger than six years and those with developmental delay, chest wall or spinal deformities, or chronic systemic illnesses were excluded.

After appropriate training in spirometry technique, pulmonary function testing was performed using a calibrated computerized spirometer in accordance with American Thoracic Society/European Respiratory Society 2019 [10] guidelines. At least three acceptable and reproducible maneuvers were obtained, and the highest values of forced vital capacity (FVC), FEV₁, FEV₁/FVC ratio, and peak expiratory flow rate (PEFR) were recorded. Results were expressed as percentages of predicted values using Indian reference equations adjusted for age, sex, and height. Participants were instructed to withhold short-acting bronchodilators for six hours prior to testing.

Functional capacity was assessed using the 6MWT, conducted along a straight indoor corridor under supervision. The results were compared with reference values provided by Agarwal et al. [9] for the 6MWT. Data were entered into Microsoft Excel (Microsoft Corporation, Redmond, WA, USA) and analyzed using IBM SPSS Statistics for Windows, Version 26.0 (Released 2018; IBM Corp., Armonk, NY, USA). Continuous variables were summarized as mean ± SD, and categorical variables were expressed as frequencies and percentages. Differences in pulmonary function parameters across asthma severity categories (mild, moderate, and severe) were analyzed using one-way ANOVA with Bonferroni post hoc correction. Associations between categorical variables were evaluated using the chi-square test, and correlations between asthma severity and spirometric indices were assessed using Pearson’s correlation coefficient.

All children with abnormal baseline FVC were offered repeat spirometry at three months, of whom 57 completed the follow-up assessment. A p-value <0.05 was considered statistically significant.

## Results

**Figure 1:**
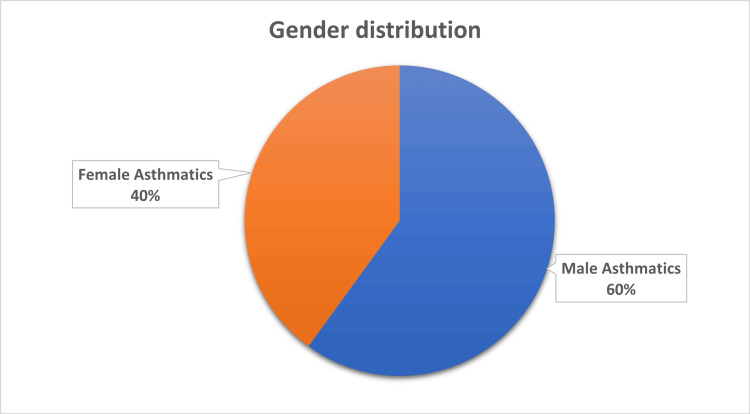
Gender distribution of study participants

A total of 250 children were enrolled in the study. Among the participants, 150 (60%) were boys and 100 (40%) were girls, yielding a male-to-female ratio of 1.5:1 (Figure 1).

Age distribution of the study sample revealed that 62 (25%), 92 (37%), and 96 (38%) children were aged 6-7, 8-9, and 10-12 years, respectively, showing a relatively balanced representation in middle childhood (Table 1).

**Table 1:** Age distribution of subjects

Age group	Frequency	Percentage
6-7 years	62	25%
8-9 years	92	37%
10-12 years	96	38%

Age distribution was broadly comparable across the three bands, with a slight predominance in the 10-12-year group.

In children with mild asthma, the majority had normal spirometric values, while small proportions had reduced FVC or PEFR. Almost all participants were able to complete the 6MWT (Table 2).

**Table 2:** Lung function parameters in mild asthma (n = 128) 6MWT, six-minute walk test; FVC, forced vital capacity; PEFR, peak expiratory flow rate

Parameter	Normal, n (%)	Abnormal, n (%)
FVC	108 (84.0%)	20 (16.0%)
PEFR	124 (97.0%)	4 (3.0%)
6MWT	124 (97.0%)	4 (3.0%)

Moderate asthma was associated with a slightly higher percentage of decreased FVC and PEFR compared with the mild group; however, most children still exhibited preserved exercise capacity (Table 3).

**Table 3:** Lung function parameters in moderate asthma (n = 92) 6MWT, six-minute walk test; FVC, forced vital capacity; PEFR, peak expiratory flow rate

Parameter	Normal, n (%)	Abnormal, n (%)
FVC	75 (82.0%)	17 (18.0%)
PEFR	87 (95.0%)	5 (5.0%)
6MWT	88 (96.0%)	4 (4.0%)

Severe asthma was characterized by significant reductions in lung-function indices: approximately two-thirds of children had low FVC, and one-third had low PEFR. The majority of participants, however, were still able to complete the 6MWT (Table 4).

**Table 4:** Lung function parameters in severe asthma (n = 30) 6MWT, six-minute walk test; FVC, forced vital capacity; PEFR, peak expiratory flow rate

Parameter	Normal, n (%)	Abnormal, n (%)
FVC	10 (33.3%)	20 (66.7%)
PEFR	20 (66.7%)	10 (33.3%)
6MWT	28 (93.3%)	2 (6.7%)

Comparative analysis showed no significant difference in FVC between mild and moderate asthma. Significant differences in the distribution of abnormal FVC values were observed between moderate versus severe and mild versus severe asthma groups (Table 5).

**Table 5:** Comparison of lung function (FVC) between asthma categories FVC, forced vital capacity

Comparison (FVC)	χ² value	p-Value	Significance
Mild vs. moderate	0.0002	0.9877	Not significant
Moderate vs. severe	4.4107	0.0357	Significant
Mild vs. severe	4.4980	0.0339	Significant

Three-month follow-up demonstrated that lung-function parameters improved in most children with mild and moderate asthma, whereas only a small proportion of children with severe asthma showed recovery (Table 6).

**Table 6:** Follow-up outcomes after three months (n = 57, abnormal FVC) FVC, forced vital capacity

Asthma severity	Improved, n (%)	Not improved, n (%)
Mild (n = 20)	12 (62.0%)	8 (38.0%)
Moderate (n = 17)	9 (52.9%)	8 (47.1%)
Severe (n = 20)	3 (15.0%)	17 (85.0%)

All children who completed the scheduled reassessment and had abnormal baseline FVC were offered follow-up spirometry at three months.

## Discussion

The present study evaluated pulmonary function and functional exercise capacity in children with asthma and demonstrated a clear stepwise worsening of spirometric impairment with increasing disease severity, with the greatest reductions observed in children with severe asthma. In contrast, six-minute walk performance remained relatively stable, showing minimal variation across severity categories. This finding highlights an important dissociation between objectively measured airflow limitation and functional ambulatory capacity in pediatric asthma. Consistent with previous evidence, our results indicate that spirometric indices provide a strong correlation with disease severity and aid in the clinical assessment of asthma and treatment planning. Chereches-Panta et al. demonstrated that asthma control is closely associated with reductions in FEV₁, FVC, and mid-expiratory flow rates, which may reveal physiological dysfunction even in children with minimal symptoms [3]. Similar longitudinal studies have shown that delayed diagnosis or underdiagnosis may result in progressive deterioration of lung volumes, particularly FEV₁ and FVC.

The high prevalence of abnormal spirometry observed in our severe asthma subgroup is consistent with findings from cohorts of uncontrolled or treatment-resistant asthma. Yimlamai et al. reported that 58% of treated school-aged children exhibited persistent airflow limitation or bronchodilator responsiveness despite ongoing controller therapy, reflecting sustained airway inflammation and early remodeling [11]. Paracha et al. further demonstrated substantial discordance between symptoms, treatment adherence, and lung function in children managed in primary care, underscoring the limited reliability of symptom-based assessments [12].

The preservation of 6MWT performance observed in our cohort aligns with previous studies suggesting that the 6MWT, when used in isolation, lacks sensitivity for distinguishing asthma severity. Anandi et al. reported that symptomatic improvement may precede measurable spirometric recovery, with functional capacity often remaining stable despite underlying airway dysfunction [13]. Similarly, intervention and self-management studies reviewed by Almutairi et al. have shown that improvements in symptoms and daily functioning can occur despite modest changes in spirometric parameters [14].

From a mechanistic perspective, the progressive reductions in FVC and PEFR across severity categories likely reflect increasing small-airway involvement, heightened bronchial hyperresponsiveness, and gradual airway narrowing. Small-airway dysfunction has emerged as an early and clinically meaningful risk marker, with reduced mid-maximum expiratory flow predicting poor disease control and future exacerbations [1]. The relative preservation of 6MWT performance may indicate that submaximal functional capacity remains intact until airflow limitation becomes substantial, or that children adapt behaviorally by unconsciously pacing their physical activity.

Rhee and Katchamat have described the phenomenon of symptom habituation and under-perception, particularly of nocturnal symptoms, as contributing to the under-recognition of physiological deterioration in pediatric asthma through behavioral compensation [15]. These observations reinforce the complementary roles of spirometry and functional assessment in childhood asthma management. Spirometric indices such as FEV₁, FVC, and PEFR provide objective quantification of airway obstruction and often detect disease progression earlier than symptom reporting. Functional assessments such as the 6MWT offer clinically relevant insights into everyday activity tolerance but should not be used in isolation to determine disease severity. When integrated, these tools contribute to a more comprehensive clinical assessment and may inform treatment modification, referral to rehabilitation services, and follow-up strategies, particularly in moderate-to-severe disease or when treatment adherence is uncertain, as demonstrated in the ARCA cohort, where longitudinal monitoring linked adherence patterns with symptom control and health-related quality of life [16].

We acknowledge that asthma severity terminology has evolved in later GINA updates; however, reclassification was not feasible as data collection was completed in 2021. The strengths of the present study include broad representation across asthma severity categories, the use of standardized spirometry and 6MWT protocols, and the integration of both physiological and functional assessments. While symptom-based classification reflects clinical control, spirometry provides objective evidence of disease progression, and both should be interpreted together.

Our study has several limitations. Physiological responses during the 6MWT, such as heart rate, oxygen saturation, and symptom scoring, were not recorded. More sensitive small-airway indices beyond mid-expiratory flow measurements were not assessed, and the single-center design limits generalizability. In addition, treatment adherence and inhaler technique, known modifiers of lung function, were not formally evaluated.

## Conclusions

Our findings indicate that spirometric impairment progresses with increasing asthma severity, whereas functional walking capacity remains relatively preserved until later stages. Response to therapy decreases as asthma severity increases, with lung function particularly compromised in children with severe asthma, who also show limited treatment response. Integrated monitoring strategies combining objective lung-function testing, clinical evaluation, and longitudinal follow-up are essential for optimal pediatric asthma management. Future research should investigate the prognostic utility of combined spirometry-exercise indices and evaluate interventions aimed at preventing or mitigating early physiological decline.
